# Biostimulation of tomato growth and biocontrol of *Fusarium* wilt disease using certain endophytic fungi

**DOI:** 10.1186/s40529-022-00364-7

**Published:** 2022-12-09

**Authors:** Amer M. Abdelaziz, Mohamed H. Kalaba, Amr H. Hashem, Mohamed H. Sharaf, Mohamed S. Attia

**Affiliations:** Botany and Microbiology Department, Faculty of Science, Al-Azhar University, Nasr City, Cairo, 11884 Egypt

**Keywords:** Tomato, *Fusarium*, Endophytic, Antioxidant enzymes, Isozymes, Resistance

## Abstract

**Background:**

Tomato plant (*Solanum lycopersicum* L.) suffers from numerous fungal pathogens that cause damage to yeild production qualitatively and quantitatively. One of the most destructive disease of tomato is *Fusarium* wilt that caused by soil borne fungus called *F. oxysporum*.

**Methods:**

In this study, the anti-*Fusarium* capabilities of the foliar application of fungal endophytes extracts have been investigated on tomato under *Fusarium* challenges. Antifungal assay, inhibition of conidial germination, disease severity, photosynthetic pigments, osmolytes, secondary metabolites, oxidative stress, peroxidase (POD) and polyphenol oxidases (PPO) isozymes were tested for potential resistance of tomato growing under *Fusarium* infection.

**Results:**

Ethyl acetate extracts of *A. flavus* MZ045563, *A. fumigatus* MZ045562 and *A. nidulans* MZ045561 exhibited antifungal activity toward *F. oxysporum* where inhibition zone diameters were 15, 12 and 20 mm, respectively. Moreover, extracts of all fungal isolates at concentration 7.5 mg/mL reduced conidia germination from 94.4 to 100%. *Fusarium* infection caused a destructive effects on tomato plant, high severity desiese index 84.37%, reduction in growth parameters, photosynthetic pigments, and soluble protein. However, contents of proline, total phenol, malondialdehyde (MDA), hydrogen peroxide (H_2_O_2_) and antioxidant enzymes activity were increased in tomato plants grown under *Fusarium* wilt. Treatment of healthy or infected tomato plants by ethyl acetate fungal extracts showed improvements in morphological traits, photosynthetic pigments, osmolytes, total phenol and antioxidant enzymes activity. Besides, the harmful impacts of *Fusarium* wilt disease on tomato plants have also been reduced by lowering MDA and H_2_O_2_ levels. Also, treated tomato plants showed different responses in number and density of POD and PPO isozymes.

**Conclusion:**

It could be suggested that application of ethyl acetate extracts of tested fungal endophytes especially combination of *A. flavus, A. nidulans* and *A. fumigatus* could be commercially used as safe biostimulation of tomato plants as well as biofungicide against tomato *Fusarium* wilt disease.

## Introduction

Agricultural crops suffered from many risks as biotic stresses including fungal pathogens that are increasing day by day in light of climatic extremes (Hashem et al. 2021). In addition to plant pathogens whose virulence is increasing and leading to severe damages that lead to crop failure and the formation of microbial toxins inside the final product, which harms the life of the final consumer (Jackson and Taylor 1996; Khalil et al. 2019; Abdelaziz et al. 2022a).The Solanaceae family is exposed to many pathogens that cause decreasing in productivity. Tomato is a short-lived perennial cropped as annual, and it is part of the Solanaceae and is usually grown for its edible fruits. Tomato considers among the important crops grown over the world for their economic and nutritional value. Tomato is an economical plant widely cultivated all over the world (Olatunji and Afolayan 2018). *Fusarium* is one of the most common fungi and is found in agricultural soils, whether they are cultivated organically or conventionally thus it classified as soil borne fungus (Elmholt 1996; Abdelaziz et al. 2022b). Tomato crops are being reduced all over the world, including Egypt, due to soil-borne diseases such as *F. oxysporum*, *Alternaria* that cause significant losses in productivity quantity and quality (Attia et al.2022a, b, c). There are many biological, natural and chemical inducers can Induce plant resistance against biotic stresses (Abdelaziz et al. 2022c; Khattab et al. 2022).

Plant growth promoting fungi (PGPF) applied as effective natural control against phytopathogens including *F. oxysporum* and improved the tomato plant growth through encourage biochemical resistance and improve the effectiveness of tomato resistance against phytopathogens including *Fusarium* spp. (Hyakumachi 2013). This biochemical defence activated by certain fungal metabolites as HCN, IAA, siderophore and by increasing plant ability to solubilize phosphate (Chakraborty et al. 2006; Attia et al. 2022a, b, c). Endophytes are microorganisms that exist in living healthy plant tissues without producing any diseased symptoms to their host plants. Endophytic fungi are very important type of endophytes that hyperdiverse (Wani et al. 2015; Khalil et al. 2021). Recently, scientists have received much attention about isolation of endophytes and the study of their natural products. plants harbor a numerous of endophytic microorganisms, owning huge metabolic diversity and varied bioactive substances that can be applied as a therapeutic toll against human pathogens as well as biotic and a biotic stresses of plants (Nisa et al. 2015, Strobel, 2018, Aldinary et al. 2021, Badawy et al. 2021, Hashem et al. 2022). Ethyl acetate extract derivative from endophytic fungi have a great activity against diverse pathogenic microorganisms due to the occurrence of active secondary metabolites including steroids, flavonoids, terpenoids, peptides, quinones, lignans, alkaloids, phenylpropanoids, phenolics and isocoumarins (Elghaffar et al. 2022).

This study aims to evaluate the anti-fungal capabilities of ethyl extracts of endophytic fungi (*A. flavus, A. nidulance, A. fumigatus*) on tomato plants under *Fusarium* infection and evaluate the promotion activity of healthy tomato plants through a safe and ecofriendly method.

## Materials and methods

### Source of endophytic fungi

Endophytic fungi isolated from healthy *Ocimum Basilicum* then identified as *A. flavus* MZ045563*, A. fumigatus* MZ045562 and *A. nidulans* MZ045561 in our previous study (Sharaf et al. 2022)**.**

### Source of the fungal pathogen

*F.oxysporum f. sp. Lycopersici* RCMB008001 was purchased from Regional Center for mycology at Al-Azhar University (RCMB) then was established by pathogenicity test according to Hibar et al. (2007). The inoculum was prepared according to Büttner et al. (2004).

### Extraction of bioactive compounds from fungi

Endophytic fungi were cultured in potato dextrose broth medium (PDB) (Oxoid) at 27 ℃ ± 2 ℃ for 21 days under static conditions. The fermentation broth was subjected to filtration under septic conditions to remove fungal mycelia. Culture filtrates of the isolated fungal endophytes were extracted twice using ethyl acetate (EtOAc) (1:1); 100 mL from each filtrate was mixed with 100 mL of ethyl acetate and placed on a vortex shaker for 10 min and settled down for 5 min until the two clear separate layers were formed. The organic layer (EtOAc) was separated from the aqueous layer by the separating funnel. The collected organic phase was evaporated under reduced pressure at 40–45 ℃ using a rotary evaporator (Heidolph VV2001, Germany); DMSO at 1 mg/mL of concentration was used to dissolve the fungal crude extract and then stored at – 20 ℃ until further experiments (Supaphon et al. 2013).

### Antifungal assay

The antifungal activity of crude extract of fungal isolates was investigated on potato dextrose agar (PDA) against *F. oxysporum* according to Sharaf et al. (2022)**.** The conidia of *F. oxysporum* were cultivated on the surface of PDA medium and incubated at 25 °C for 10 days. Then, the culture of *F. oxysporum* was surface flooded with 10 mL of sterilized water to obtain the suspension of fungal conidia which adjusted to around 10^5^ conidia/mL by diluting and counting. Sterile Petri dishes (120 mm) containing PDA media were inoculated with 100 µL of *F. oxysporum* conidial suspension. Then agar wells (8 mm) were cut using sterile cork borer and loaded with 100 µL of the crude extract of fungal isolates (5 mg/mL) as well as fluconalzole (25 µg/well) which used as antifungal control. After incubation of inoculated PDA plates at 25 °C for 5 days and the resulted inhibition zone diameter (mm) were measured and represented as mean ± standard error values. The experiment was performed in triplicates.

### Inhibition of conidial germination assay

A suspension of 10 days old *F. oxysporum* conidia was prepared as previously mentioned in the antifungal assay. In this assay, the effects of endophytic fungi crude extracts on *F. oxysporum* conidial germination were examined at varieing concentrations (2.5, 5 and 7.5 mg/mL) as performed by Rongai et al. (2012) with some modifications. Each well of the microtiter plate received 200 μL of a combination containing: 80 μL of conidial suspension, 100 μL of double strength PDB, and 20 μL of the tested fungal crude extract. Each treatment was carried out in three wells, with one plate row containing an untreated spore suspension in PDB as a negative control. Conidial germination was determined by mounting 10 μL samples of each treatment on a hemocytometer slide after 24, 48, and 72 h and counting the number of germinated and non-germinated spores in five squares at 200 × magnification. The percentage of germination was determined and averaged for the three wells using the following equation:

$$\mathrm{Spore\, germination}\, \left(\%\right)\,= \frac{\mathrm{Germinated\, spores } }{\mathrm{Total\, spores}} \times 100$$

### Pot experiment

Four weeks of age tomato seedlings were obtained from the Agricultural Research Center (ARC), Giza, Egypt. Uniform seedlings were transplanted into pots (40 cm in diameter) contain a mixture of sand and clay (1:3 W/W), total 8 kg, in a plastic greenhouse at experimental plant garden of Botany and Microbiology Department, Faculty of Science, Al-Azhar University, Egypt. The pots were coordinated in a completely randomized design with 8 replicates for each treatment. Pots were arranged as follows; T1-Healthy control (sowing tomato seedlings in sterilized soil), T2-Infected control (sowing the tomato seedlings in sterilized soil inoculated with *F. oxysporum*), T3-Healthy plants treated with *A. flavus*, T4-Healthy plants treated with *A. nidulan*s, T5-Healthy plants treated with *A. fumigatus*, T6-Healthy plants treated with combination of *A. flavus, A. nidulas* and *A. fumigatus*, T7-Infected plants treated with *A. flavus* and T8-Infected plants treated with *A. nidulans*, T9-Infected plants treated with *A. fumigatus*,T10-Infected plants treated with combination of *A. flavus, A. nidulans* and *A. fumigatus*. For plant resistance evaluation biochemical signals from plant samples were analyzed 45 days after sowing, and the disease was assayed.

### Disease symptoms and disease index

The disease symptoms were observed 45 days after sowing and the disease index and plant protection were assessed using a score consisting of five classes, as described in Elbasuney et al. (2022) with minor modifications; (1) minor yellowing of lower leaves, (2) moderate yellow plant, (3) wilted plant with browning of vascular bands, and (4) severely stunted and damaged plants. The percent disease index (PDI) was determined using a five-grade scale and the formula: PDI = (1n_1_ + 2n_2_ + 3n_3_ + 4n_4_)100/4n_t_, where n_1_-n_4_ represents the number of plants in each class and nt represents the total number of plants examined. In addition, the following formula was used to obtain % Protection (P %): P % = (A–B/A) X100, where A is the PDI in infected control plants and B is the PDI in infected plants treated with fungal endophytes.

### Photosynthetic pigment determination

A former procedure mentioned in the study Cohen-Bazire et al. (1966) was used to assess the existence of chlorophyll a (Chl a), chlorophyll b (Chl b) and carotenoids in fresh leaves. Throughout this technique, photosynthetic pigments were extracted from fresh leaves (0.5 g) using 50 mL of acetone (80%) then the green color was determined spectrophotometrically at 665, 649, and 470 nm after the extract was filtered.

### Determination of the content of osmolytes

The soluble sugar content of the dried shoot was calculated by the method described by Irigoyen et al. (1992). The dried shoots (0.5 g) from each treatments were diluted with 5 mL of 30% trichloroacetic acid (TCA) and 2.5 mL of 2% phenol and filtered through filter paper, then 1 mL of the filtrate was treated with 2 mL of anthrone reagent (2 g anthrone/L of 95% H_2_SO_4_). 620 nm was used to determine the produced blue-green color.

The procedure of El-Tayeb (2005) was used to determine the soluble protein content of the dry shoot.One mL of this extract was combined with 5 mL of alkaline reagent (50 mL of 2% Na_2_CO_3_ prepared in 0.1 N NaOH and 1 mL of 0.5% CuSO_4_ prepared in 1% potassium sodium tartrate) and 0.5 mL of Folin’s reagent (diluted by 1:3 v/v). After 30 min, a color change could be seen at a wavelength of 750 nm.

The proline content was measured in the dry shoot according to Bates et al. (1973). The dried shoots (0.5 g) were digested by 10 mL (3%) of sulfosalicylic acid in this technique. Using a boiling water bath, 2 mL of the filtrate was mixed with 2 mL of ninhydrin acid and 2 mL of glacial acetic acid for an hour, then the mixture was placed in an ice bath to stop the reaction. 4 mL of toluene was added to the mixture, then the absorbance at 520 nm was determined.

### Determination of total phenol contents

The technique of Jagota et al. (1982) was used to estimate the ascorbic acid content of the dry shoot. Total dry shoot phenol content was measured using the Dai et al. (1993) procedure.

### Estimation of malondialdehyde and hydrogen peroxide contents

The content of MDA in fresh leaf was measured according to Hu et al. (2004). The H_2_O_2_ content of fresh leaf was measured as stated by Mukherjee and Choudhuri (1983).

### Assay of antioxidant enzymes activity

POD activity was assayed according to that method described by Bergmeyer (1974). The activity of PPO was calculated by the procedure used by Matta and Dimond (1963). The activities of POD and PPO were assayed in fresh tomato leaves.

### Isozymes electrophoresis

Native polyacrylamide gel electrophoresis (Native-PAGE) isozyme electrophoresis was performed to identify isozyme differences between control and treatment. PPO isozymes in leaves (100 mg fresh weight) samples were estimated as described by Bradford; Thipyapong, et al., (Bradford 1976; Thipyapong et al. 1995). POD in fresh leaves isozymes were assessed by the procedure defined by Barceló, et al. (1987).

### Statistical analysis

One-way variance analysis (ANOVA) applied to the resulting data. The least significant difference (LSD test) using CoStat (CoHort, Monterey, CA, USA) was used to demonstrate statistically relevant differences between treatments at p < 0.05. Results are shown as mean ± standard errors (n = 3) according to Snedecor and Cochran (1980).

## Results

### Antifungal assayof endophytic fungi

**Fig. 1:**
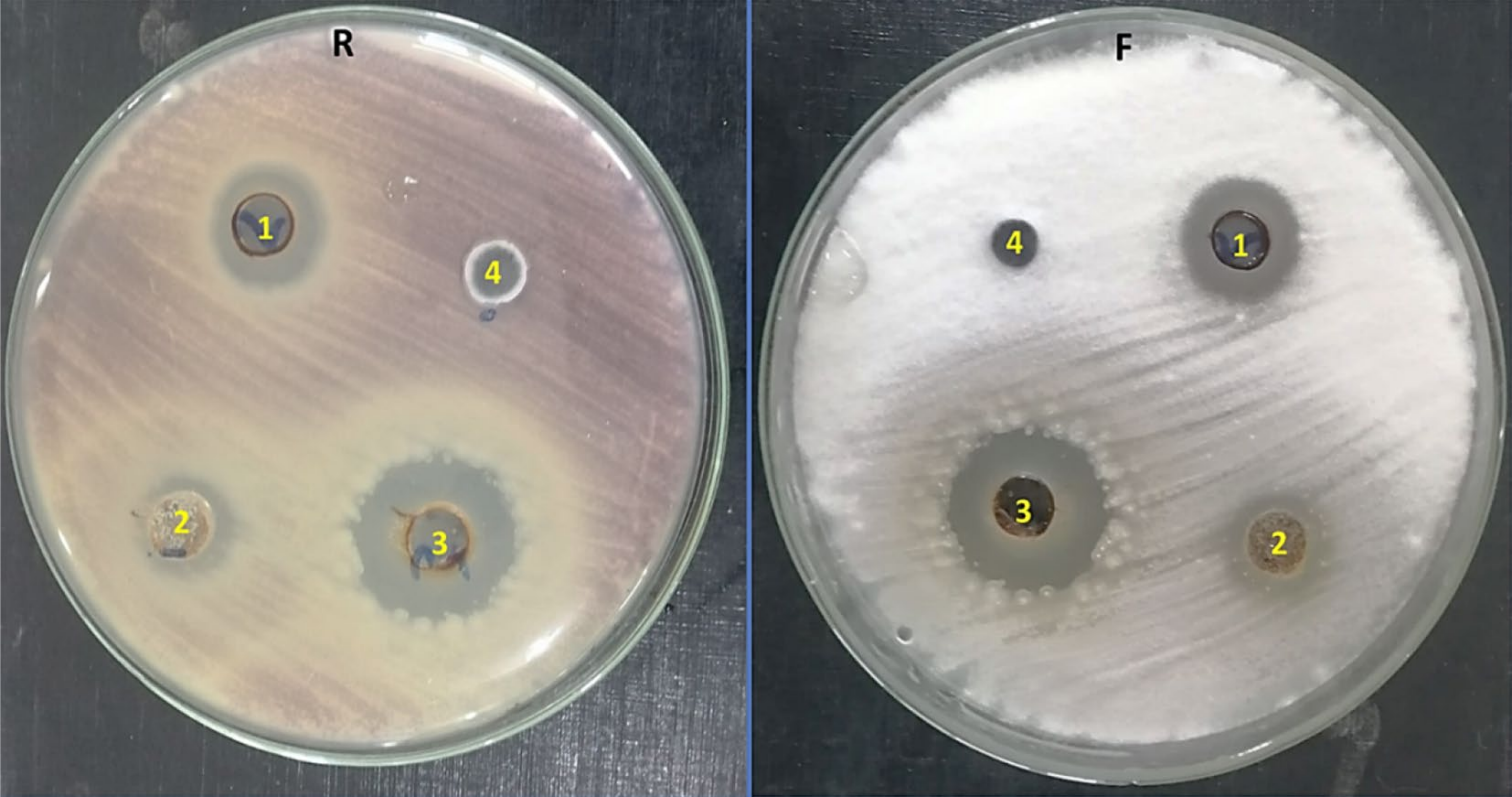
Antifungal activity of (1): *A. flavus*, (2): *A. fumigatus*, (3): *A. nidulans* crude extracts and (4): fluconazole against *F. oxysporum* using agar well diffusion method. (F = front and R = reverse)

Crude extracts of *A. flavus* MZ045563*, A. fumigatus* MZ045562 and *A. nidulans* MZ045561 were tested for antifungal activity against *F. oxysporum*. They inhibited the growth of *F. oxysporum* with inhibition zone diameters of 15.00, 12.33 and 20.83 mm respectively, whereas fluconazole had no impact (Table 1). Figure 1 illustrates some significant observations during the antifungal assay, both *A. fumigatus* and *A. nidulans* extracts had another impact further than the inhibition zones, such as absence of off white, fairly dense appearance of aerial mycelium with no production of light purple pigment in the diffusion zone of these extracts.

**Table 1:** antifungal activity of fungal ethyl acetate extracts against *F. oxysporum*

Treatment	Mean of inhibition zone diameter(mm ± SD)
*A. flavus* crude extract (T-7)	15 ± 2.00
*A. fumigatus* crude extract (T-5)	12.33 ± 0.577
*A. nidulans* crude extract (T-4)	20.83 ± 1.443
Fluconazole	0

**Fig. 2:**
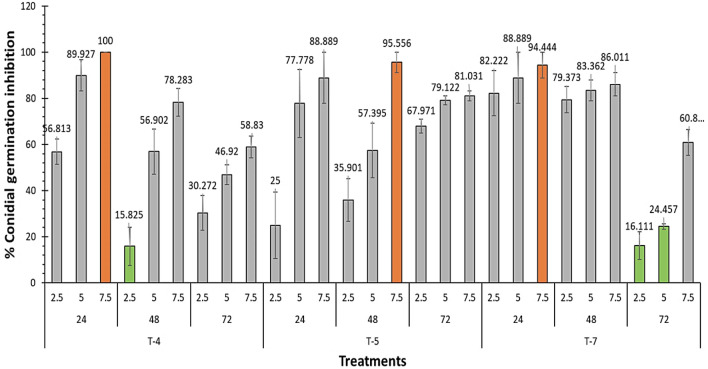
Inhibition of conidial germination of *F. oxysporum* with different concentrations of the crude extract of *A. flavus* (T-7), *A. fumigatus* (T-5), and *A. nidulans* (T-4) after 24, 48 and 72 h

Overall, the results demonstrated that fungal extracts of *A. flavus*, *A. fumigatus* and *A. nidulans* displayed antifungal efficacy by reduction of the germination of *F. oxysporum* conidia in a concentration-dependent manner. When we compared different extract at the same times, the results were show (Fig. 2) difference between them but the extracts of *A. nidulans* (T-4) followed by *A. flavus* (T-7) showed a strong effect alternately at 24 and 48 h, while the effect of *A. fumigatus* (T-5) was always the lowest at these times, while after 72 h it was mostly reduced the germination of conidia than *A. nidulans* (T-4) followed by *A. flavus* (T-7)*.* By comparing the concentrations of each extract at the same time, we observed substantial variations, and the concentration of 7.5 mg/mL exhibited most significant reduction of conidia germination, for example this concentration reduced the conidia germination by rate ranging from 94.40 to 100% after 24 h (Fig. 2).

### Disease index

The results presented in the (Table 2) indicated that *F. oxysporum* highly destructive were the percentage of disease index (PDI) of infected tomato plants (84.37%). On the other hand, all tested endophytic isolates showed a great decrease in the disease symptoms and PDI and thus give a high percentage of protection (Table 2). Treatment of infected plants with combination of *A. flavus, A. nidulance* and *A. fumigatus* and *A. nidulans* were the best treatments which reduced percent disease indexes in (12.5% and 25%) as well as high protection by (85.18% and 70.36%) respectively then followed by *A. fumigatus*, and *A. flavus* which recorded PDI (34.37% and 37.50%) and protection (59.26% and 55.55%) respectively.

**Table 2:** Effect of fungal ethyl acetate extracts on DI of infected Tomato plants

Treatments	Disease symptoms classes					DI (disease index) (%)	Protection (%)
	0	1	2	3	4		
Control infected (T2)	0	0	1	3	4	84.37	0
Infected + *A. flavus* (T7)	2	2	3	0	1	37.50	55.55
Infected + *A. nidulans* (T8)	3	3	1	1	0	25.00	70.36
Infected + *A. fumigatus* (T9)	2	2	3	1	0	34.37	59.26
Combination of *A. flavus, A. nidulans* and *A. fumigatus* (T10)	5	2	1	0	0	12.50	85.18

### Photosynthetic pigments

**Fig. 3:**
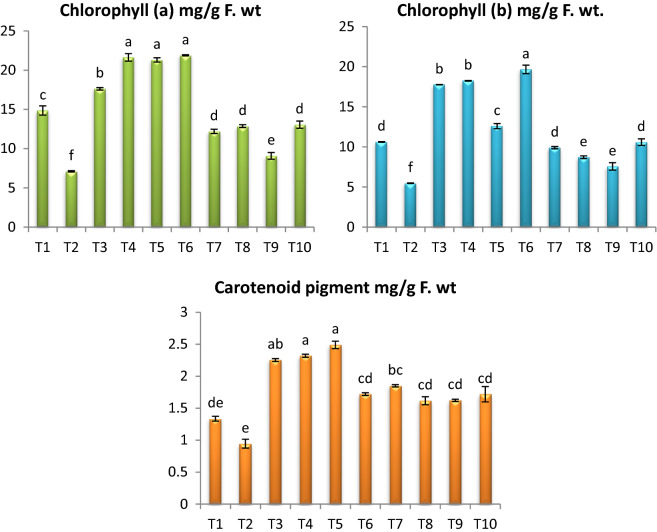
Effect of *F. oxysporum* and endophytic isolates (*A. flavus, A. nidulans* and *A. fumigatus*) individual or combination on photosynthetic pigments of tomato plants. Data presented as means ± SD (n = 3). Data followed by different letters (a:f) are significantly different LSD test at P ≤ 0.05. FW: fresh weight. ( T1-Healthy control; T2-Infected control; T3-Healthy treated with *A. flavus*; T4-Healthy treated with *A. nidulance*; T5-Healthy treated with *A. fumigatus*; T6-Healthy treated with combination of *A. flavus, A. nidulance* and *A. fumigatus*; T7-Infected treated with *A. flavus*; T8-Infected treated with *A. nidulance*; T9-Infected treated with *A. fumigatus* and T10-Infected treated with combination of *A. flavus, A. nidulance* and *A. fumigatus*

The contents of chlorophyll a and b were highly significantly decreased in infected plants (Fig. 3). However infected plants treated with endophytic isolates (*A. flavus, A. nidulans* and *A. fumigatus*) individual or combination showed a significant increase when compared with infected plants. Furthermore, combination of *A. flavus, A. nidulans* and *A. fumigatus* then *A. nidulans* showed a significant increase in the contents of Chl a (83.68% and 81.06%) but combination of *A. flavus, A. nidulans* and *A. fumigatus* then *A. flavus* resulted to a significant increase in Chl b by (93.67% and 81.10%) respectively. The healthy tomato plants treated with with endophytic isolates (*A. flavus, A. nidulans* and *A. fumigatus*) singular or mixture showed a significant increase in chlorophyll a and b. On the other hand, in infected plants the content of carotenoids decreased by 42.02% when being compared with healthy plants. Moreover, the obtained results illustrated that in *Fusarium* infected plants the content of carotenoids was increased in response to the treatment with endophytic isolates (*A. flavus, A. nidulans* and *A. fumigatus*) individual or combination.

### Osmolytes

**Fig. 4:**
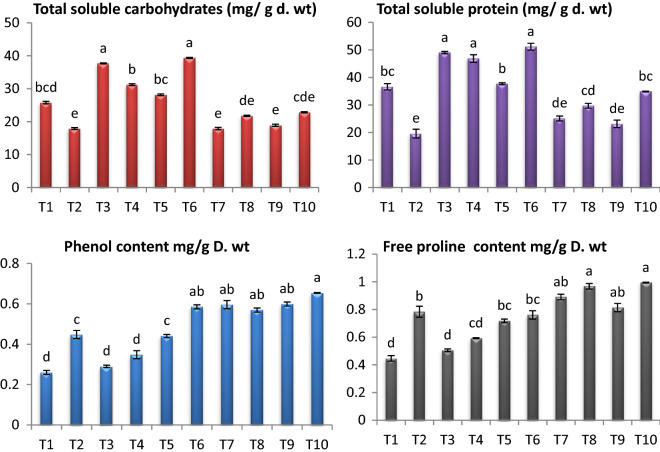
Effect of *F. oxysporum* and endophytic isolates on osmolytes (mg/g dry weight) of tomato plants. Data presented as means ± SD (n = 3). Data followed by different letters (a:f) are significantly different LSD test at P ≤ 0.05. FW: fresh weight. (T1-Healthy control; T2-Infected control; T3-Healthy treated with *A. flavus*; T4-Healthy treated with *A. nidulance*; T5-Healthy treated with *A. fumigatus*; T6-Healthy treated with combination of *A. flavus, A. nidulance* and *A. fumigatus*; T7-Infected treated with *A. flavus*;T8-Infected treated with *A. nidulance*; T9-Infected treated with *A. fumigatus* and T10-Infected treated with combination of *A. flavus, A. nidulance* and *A. fumigatus*)

*Fusarium* infected tomato plants showed decrease in contents of soluble charbohydrates and soluble proteins by 53.82% and 46.43% respectively (Fig. 4). On the other hand, the content of proline and phenols were increased significantly by 75.39% and 72.30% as compared to healthy control plants. Application of endophytic isolates (*A. flavus, A. nidulans* and *A. fumigatus*) enhanced the contents of soluble charbohydrates, soluble proteins, prolin and phenols in shoots of infected tomato plants compred to infected control. The highest recorded increase in contents of soluble sugars, soluble proteins, proline and protein content was noticed in combination of *A. flavus, A. nidulans* and *A. fumigatus* by 92.015%, 77.85%, 26.78% and 45.90% respectively over *Fusarium* infected plants. Application of endophytic isolates *A. flavus, A. nidulans* and *A. fumigatus* individual or combination on healthy plants elevated the contents of soluble charbohydrates, soluble proteins, prolin content and phenols respectively over healthy control plants (Fig. 4). The highest recorded increase in response to application of tested endophytic isolates on healthy plants were in the case of Combination of *A. flavus, A. nidulans* and *A. fumigatus*.

### Oxidative stress

**Fig. 5:**
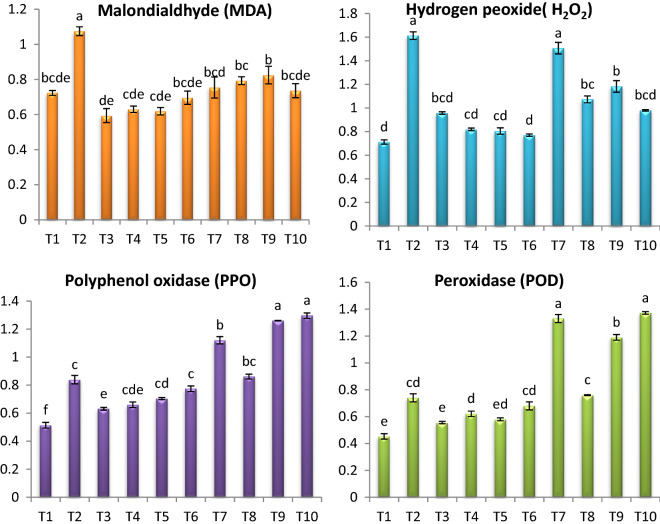
Effect of *F. oxysporum* and endophytic isolates on (MDA) (nmol g^−1^ fresh weight); (H_2_O_2_) (mg g^−1^ fresh weight); POD (unit/g) f. wt./h. and PPO(unit/g) f. wt./ h. of tomato plants. Data presented as means ± SD (n = 3). Data followed by different letters are significantly different LSD test at P ≤ 0.05. (T1-Healthy control; T2-Infected control; T3-Healthy treated with *A. flavus*; T4-Healthy treated with *A. nidulance*; T5-Healthy treated with *A. fumigatus*; T6-Healthy treated with combination of *A. flavus, A. nidulance* and *A. fumigatus*; T7-Infected treated with *A. flavus*; T8-Infected treated with *A. nidulance*; T9-Infected treated with *A. fumigatus* and T10-Infected treated with combination of *A. flavus, A. nidulance* and *A. fumigatus*)

*Fusarium* infection caused accumulation the contents of MDA and H_2_O_2_ by 48.86% and 95.99% respectively, comparing to healthy tomato plants (Fig. 5). The content of MDA was dropped in response to different endophytic isolates treatments by 31.53%, 29.86%, 26.23%, and 23.25% at combination of *A. flavus, A. nidulans* and *A. fumigatus, A. flavus, A. nidulance* and *A. fumigatus* respectively. The application of endophytes singular or mixure decreased the MDA of healthy tomato plant. While the content of H_2_O_2_ was decreased by 39.24%, 33.47%, 26.65%, 6.57% at combination of *A. flavus, A. nidulans* and *A. fumigatus*, *A. nidulans*, *A. fumigatus*, and *A. flavus*, respectively comparing to infected control (Fig. 5). The application of endophytes odd or mixure increased the H_2_O_2_ of healthy tomato plant. The highest value of H_2_O_2_ recorded by *A. flavus*. The activity of POD and PPO were boosted in *Fusarium* infected tomato plants comparing to healthy (Fig. 5). Moreover, application of all tested endophytic isolates were increased the activity of POD and PPO. The highest recorded increase in POD and PPO activity in response to application of tested endophytic isolates on infected plants was in the case of Combination of *A. flavus, A. nidulans* and *A. fumigatus*. On the other hand, application of endophytes increased the POD and PPO activities of healthy tomato plants. The most effective treatment was combination of *A. flavus, A. nidulans* and *A. fumigatus*.

### Isozymes

#### Peroxidase (POD) isozymes

**Fig. 6:**
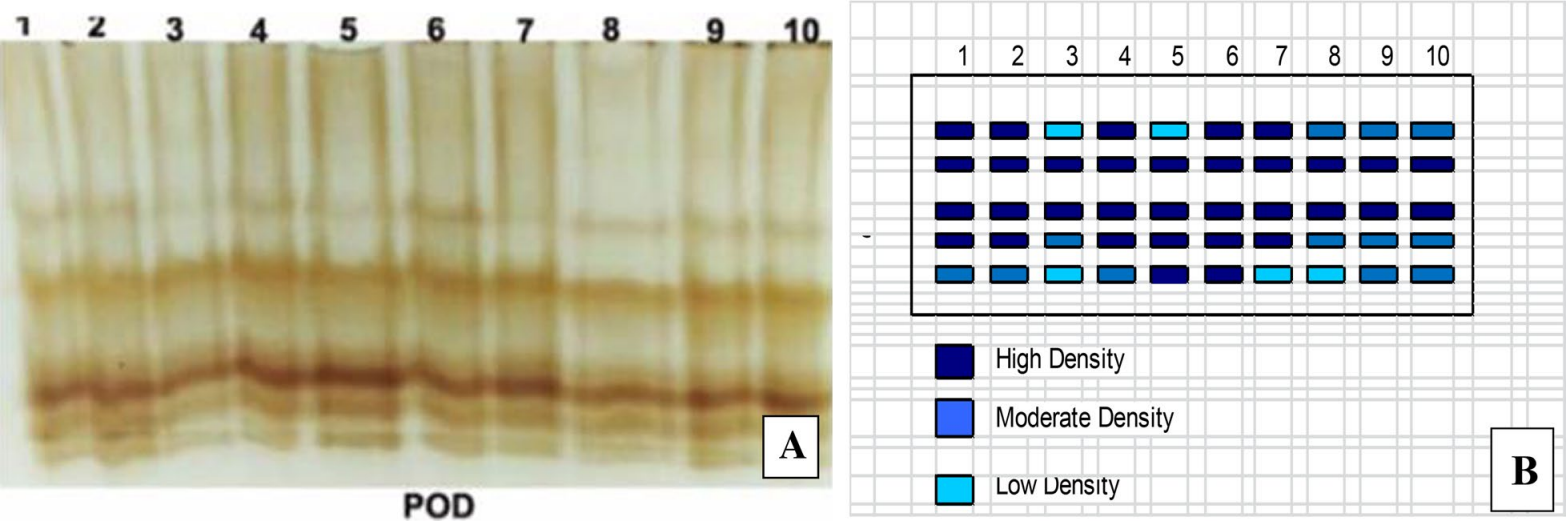
Effect of *F. oxysporum* and endophytic isolates on peroxidase isozyme of tomato plants. **A** POD, **B** Ideogram analysis of POD isozyme (T1-Healthy control; T2-Infected control; T3-Healthy treated with *A. flavus*; T4-Healthy treated with *A. nidulance*; T5-Healthy treated with *A. fumigatus*; T6-Healthy treated with combination of *A. flavus, A. nidulance* and *A. fumigatus*; T7-Infected treated with *A. flavus*; T8-Infected treated with *A. nidulance*; T9-Infected treated with *A. fumigatus* and T10-Infected treated with combination of *A. flavus, A. nidulance* and *A. fumigatus*)

Native PAGE in (Fig. 6) and (Table 3) appered five POD isozymes at Rf (0.378, 0.568, 0.795, 0.849 and 0.908). *Fusarium*-infected plants showed greatly overexpressed POD that showed 5 bands including one faint band, three moderate bands and one highly dense band. Control healthy plants expressed the lowest POD expression that they produced 3 faint bands and tow moderate band. The highest recorded increase in POD expressed in response to application of tested endophytic isolates on infected plants was in the case of combination of *A. flavus, A. nidulans* and *A. fumigatus*; *A. fumigatus*; *A. flavus* and finally *A. nidulans* respectively.

**Table 3:** Isomers of peroxidase enzymes and their retention factor (Rf) in response to *F. oxysporum* and endophytic isolates on tomato plants

Peroxidase groups	Relative mobility	1	2	3	4	5	6	7	8	9	10
Px1	0.3	1^++^	1^−^	1^++^	1^++^	1^−^	1^++^	1^+^	1^+^	1^+^	1^+^
Px2	0.45	1^++^	1^++^	1^++^	1^++^	1^++^	1^++^	1^++^	1^++^	1^++^	1^++^
Px3	0.7	1^++^	1^++^	1^++^	1^++^	1^++^	1^++^	1^++^	1^++^	1^++^	1^++^
Px4	0.8	1^++^	1^++^	1^+^	1^++^	1^++^	1^++^	1^++^	1^+^	1^+^	1^+^
Px5	0.85	1^+^	1^+^	1^−^	1^+^	1^++^	1^++^	1^−^	1^−^	1^+^	1^+^

Where, 1 present band, 0 Absent band, +  + High density, + Moderate density and − Low density (T1-Healthy control; T2-Infected control; T3-Healthy treated with *A. flavus*; T4-Healthy treated with *A. nidulance*; T5-Healthy treated with *A. fumigatus*; T6-Healthy treated with combination of *A. flavus, A. nidulance* and *A. fumigatus*; T7-Infected treated with *A. flavus*; T8-Infected treated with *A. nidulance*; T9-Infected treated with *A. fumigatus* and T10-Infected treated with combination of *A. flavus, A. nidulance* and *A. fumigatus*)

#### Polyphenoloxidase (PPO) isozymes

**Fig. 7:**
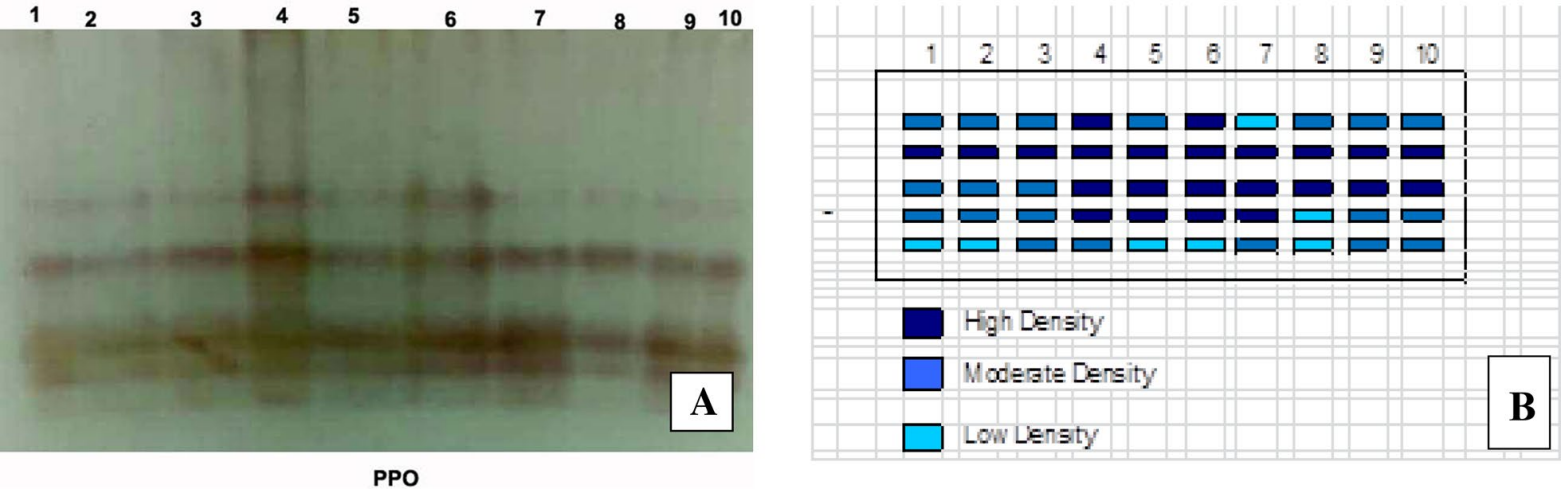
Effect of *F. oxysporum* and endophytic isolates on PPO isozyme of tomato plants. **A** PPO, **B** Ideogram analysis of PPO isozyme (T1-Healthy control; T2-Infected control; T3-Healthy treated with *A. flavus*; T4-Healthy treated with *A. nidulance*; T5-Healthy treated with *A. fumigatus*; T6-Healthy treated with combination of *A. flavus, A. nidulance* and *A. fumigatus*; T7-Infected treated with *A. flavus*; T8-Infected treated with *A. nidulance*; T9-Infected treated with *A. fumigatus* and T10-Infected treated with combination of *A. flavus, A. nidulance* and *A. fumigatus*)

The polyphenol oxidase isozyme of tomato plant leaves showed five PPO isozymes at Rf (0.391, 0.528, 0.724, 0.809 and 0.876) (Fig. 7) and (Table 4).Untreated Infected plants showed the highly PPO expression that produced 4 bands including three moderate bands and one faint band when compared with Untreated control healthy plants that expressed the lowest PPO that produced 3 faint bands. Under *Fusarium* infection conditions it was found that the the highest recorded increase in PPO expressed in response to application of tested endophytic isolates on infected plants was in the case of combination of *A. flavus, A. nidulance* and *A. fumigatus* then *A. flavus, A. nidulance* and finally *A. fumigatus* respectively.

**Table 4:** Isomers of polyphenoloxidase (PPO) izoenzymes and their Retention factor (Rf) in response to *F. oxysporum* and endophytic isolates

Peroxidase groups	Relative mobility	1	2	3	4	5	6	7	8	9	10
PPO1	0.3	1^+^	1^+^	1^+^	1^++^	1^+^	1^++^	1^−^	1^+^	1^+^	1^+^
PPo2	0.45	1^++^	1^++^	1^++^	1^++^	1^++^	1^++^	1^++^	1^++^	1^++^	1^++^
PPO3	0.7	1^+^	1^+^	1^+^	1^++^	1^++^	1^++^	1^++^	1^++^	1^++^	1^++^
PPO4	0.8	1^+^	1^+^	1^+^	1^++^	1^++^	1^++^	1^++^	1^−^	1^+^	1^+^
PPO4	0.85	1^−^	1^−^	1^+^	1^+^	1^−^	1^−^	1^+^	1^−^	1^+^	1^+^

Where, 1 present band, 0 Absent band, +  + High density + Moderate density and − Low density (T1-Healthy control; T2-Infected control; T3-Healthy treated with *A. flavus*; T4-Healthy treated with *A. nidulance*; T5-Healthy treated with *A. fumigatus*; T6-Healthy treated with combination of *A. flavus, A. nidulance* and *A. fumigatus*; T7-Infected treated with *A. flavus*; T8-Infected treated with *A. nidulance*; T9-Infected treated with *A. fumigatus* and T10-Infected treated with combination of *A. flavus, A. nidulance* and *A. fumigatus*)

## Discussion

*Fusarium* wilt disease is considered one of the greatest serious challenges facing countries, especially Egypt. Crops are being destroyed all over the world, including Egypt by soil-borne diseases such as *F. oxysporum* that cause great losses in terms of quantity and quality (Abdelaziz et al. 2021; Attia et al. 2021a, b). Therefore, the study of environmental influences and pathogenic factors that are increasing in severity makes it important to search for safe and easy-to-use alternatives with high efficiency against plant pathogens (Adnan et al. 2019; Chen 2020). *F. oxysporum* produces mycelia, conidia, and chlamydospores that play a role during host infection, producing damaging vascular wilts, rots and damping-off diseases (Doohan and Zhou 2017; Srinivas et al. 2019). However, in this study, endophytic fungal extracts had the ability to inhibit *F. oxysporum* growth as well as the formation of both aerial hyphae and pigmentation outside of the inhibitory zones. Furthermore, these extracts greatly reduced the germination of *F. oxysporum* conidia. The crude extracts of endophytic fungi under investigation include antifungal compounds such as 9-Octadecenoic acid (Z) methyl ester, methyl stearate, 9,17-Octadecadienal (Z) Linoleoyl chloride, and ethyl iso-allocoholate (Sharaf et al. 2022). Endophytic aspergilli were applied as smart biological control against *Fusarium* wilt as well as induction of healthy tomato plants by enhancement the systemic resistance of the tomato plant against *Fusarium* wilt disease. Tomato plants treated with endophytic aspergilli showed improvement of the morphological, physiological, molecular traits of infected tomato plants (Dhouib et al. 2019; Abdel-Motaal et al. 2020; Aldinary et al. 2021).

Disease severity was the first guide to govern systemic resistance in treated plants by endophytic fungi. Results presented in this study reported that *F. oxysporum* shows a highly destructive effect on tomato plants that caused typical wilt symptoms with DI 84.37%, which is similar to earlier studies on the same pathogenic fungus (Sathiyabama and Charles 2015; Abdallah et al. 2016; Srinivas et al. 2019; Abdelaziz et al. 2022c). Using endophytic fungi extracts to treat *Fusarium* wilt-infected tomato plants greatly reduced the disease symptoms, which is the primary criterion for assessing resistance in the tomato plant. Results showed that treatment of infected plants with combination of *A. flavus, A. nidulans* and *A. fumigatus* and *A. nidulans* were the best treatments which reduced percent disease indexes by (12.5% and 25%) and increased protection by (85.18 and 70.36%) respectively then *A. fumigatus* and *A. flavus* which recorded PDI by (34.37% and 37.50%) and protection by (59.26% and 55.55%) respectively, these results are consistent with (Alabouvette et al. 2009; Righini and Roberti 2019).

Vegetative growth (shoot length, root length and number leaves per plant) were significantly decreased due to *F. oxysporum*. It should be noted that, the destruction of vegetative growth traits may be related to various factors; Among them are hormonal disruption, oxidative explosion, pathogen control over cell contents, and consequently high osmotic pressure (Sagi et al. 2004; Bashan and De-Bashan 2010; Kang et al. 2021). Many studies have shown that the application of endophytic fungi such as *A. alabamensis, A. tubingensis* and *A. oryzae* improved tomato morphological traits (Nefzi et al. 2019, Aldinary et al. 2021) and biostimulate tolerance of seedlings under biotic stress conditions as well as healthy plants (Khan et al. 2012; Morsy et al. 2020; Saia et al. 2021; Sonawane et al. 2022).

The improvement of the photosynthesis pigments is a strong positive evidence for the occurrence of resistance against the disease as a result of the application of endophytic fungi extracts and became one of the visible evidence. In the present study the contents of chlorophyll a and b were highly significantly decreased in infected tomato plants due to *Fusarium* infection. The severe deficiency of chlorophyll pigments may be due to a decrease in the number of leaves responsible for light capture and photosynthesis, or it may also be due to increased activities of chlorophyll-degrading enzymes (Hörtensteiner and Kräutler 2011; Rahman 2019). Results of the current study showed that treatment of infected tomato plants by endophytic fungi extracts significantly improved plant resistance by increasing photosynthetic pigments. This increase might be approved to enhanced stomatal conductance, transpiration rate and/or cell size and number (Maghsoudi et al. 2016; Ferus et al. 2019). Endophytic fungi may confer additional plant defensive mechanisms as jasmonic acid, or salicylic acid (Ownley et al. 2010; Yan et al. 2019; Poveda et al. 2020). Also it may trigger NADPH oxidase activity, thereby activating the production of H_2_O_2_ or by the antioxidant activity,thus, endophytic fungi could activate ROS scavenging systems in plants (Zou et al. 2021). The accumulation of osmolyte plays a vital role in capturing free radicals, protecting cells from oxidation, and supplying plant cells with energy (Szabados and Savouré 2010; Das and Roychoudhury 2014). Application of endophytic fungi extracts especially combination of *A. flavus, A. nidulans* and *A. fumigatus* enhanced osmolytes in shoots of tomato plants. These results are in agreement with study achieved by Aldinary et al. (2021). Endophytic fungi caused an enhancement in the contents of soluble sugars, soluble protein throughout its role in increasing the expression of enzymes involved in glycolysis (Ghaffari et al. 2019; De Rocchis et al. 2022). The accumulation of proline in the plant prevents the damage of the photosynthesis pigments by capturing the free radicals (Alnusairi et al. 2021). In this study, *Fusarium* infection increased the contents of total phenols in tomato plants. Our results are in harmony with other researchers (Baaziz 2011; Mikulic-Petkovsek et al. 2013; Abdelaziz et al. 2021). Phenols play an important role in capturing free radicals which reduces oxidative stress in cells (Murray et al. 2007). The accumulation of phenolic compounds serves as an adaptive strategy against plant disease (Daayf et al. 2012). Our study indicated that treatment with *A. flavus*, *A. fumigatus* and *A. nidulans* improved contents of phenolic compounds significantly which directly decay lipid oxidation during transporting a phenolic hydrogen atom to a radicle (Kim et al. 2007; Ćilerdžić et al. 2014). Oxidative stress caused by *F. oxysporum* led to serious disruption of plant cell and increase the contents of MDA and H_2_O_2_ in leaves of tomato plants. The content of MDA and H_2_O_2_ were dropped in response to different endophytic isolates treatments.

Moreover, Khan et al. (2012) stated that endophyte application significantly reduced the contents of MDA in stressed plants. Abdelaziz et al. (2021) reported that antioxidant enzymes POD and PPO provide a large number of defensive enzymes associated with *Fusarium* infection. These enzymes act as initial steps in increasing plant resistance to various stresses (Van Loon et al. 1998; Rios-Gonzalez et al. 2002). The results showed that the activity of POD and PPO were boosted in *Fusarium* infected tomato plants comparing to untreated control plants. Moreover, application of all tested endophytic isolates (*A. flavus, A. nidulans* and *A. fumigatus*) individual or combination resulted to increasing the activity of POD and PPO as compared with control. The plants showed different mechanisms to adaptive with stress by increase the activity of certain antioxidant enzymes to keep ROS at the lower level in the cell. POD plays an important role in elimination of H_2_O_2_ excess by bioconversion to H_2_O (Rios-Gonzalez et al. 2002). POD and PPO activities increased in infected plants as well as plants treated with tested endophytic isolates (*A. flavus, A. nidulans* and *A. fumigatus*) compared to un-treated infected plants. Isozymes are one of the key control mechanisms for cell metabolism in plants, thus changes in isozyme profiles play an important role in cellular protection against pathogens (Harb et al. 2010, Shigeoka et al. 2022). The induction of isozymes play an important role in the cellular defense against oxidative stress (El-Beltagi et al. 2010). These results reflecting the ameliorative role of endophytic isolates (*A. flavus, A. nidulans* and *A. fumigatus*) in protecting tomato plants against *Fusarium* wilt in tomato plant.

## Conclusions

Application of ethyl acetate extracts of (*A. flavus, A. nidulans* and *A. fumigatus*) ameliorate the negative impact of tomato *Fusarium* wilt through reduced PDI and enhancement of protection against pathothogen, improvement photosynthetic pigments, increasing osmoprotectant compounds and antioxidant system. So it could be used in agricultural fields especially combination of *A. flavus, A. nidulans* and *A. fumigatus*. For more, Application of *A. flavus, A. nidulans* and *A. fumigatus* singalular or combination to enhancement healthy tomato plant growth. Ethyl acetate extracts of *A. flavus, A. nidulance* and *A. fumigatus* can be applied as safe biofungicide as well as biostimulant of growth. Accordingly, endophytic fungi (*A. flavus, A. nidulans* and *A. fumigatus*) are promising isolates for potential applications in agricultural application and as smart biological control against *F. oxysporum* which infected tomato plants and causing wilt disease. In the future, these isolates should be highlighted and applied as biocides and therapeutic nutrients to reduce the spread of plant diseases and improve plant immunity).

## Data Availability

All data and materials viable**.**

## References

[CR1] Abdallah RAB, Mokni-Tlili S, Nefzi A, Jabnoun-Khiareddine H, Daami-Remadi M (2016). Biocontrol of *Fusarium* wilt and growth promotion of tomato plants using endophytic bacteria isolated from *Nicotiana glauca* organs. Biol Control.

[CR2] Abdelaziz AM, Dacrory S, Hashem AH, Attia MS, Hasanin M, Fouda HM, Kamel S, ElSaied H (2021). Protective role of zinc oxide nanoparticles based hydrogel against wilt disease of pepper plant. Biocatal Agric Biotechnol.

[CR3] Abdelaziz AM, Attia MS, Salem MS, Refaay DA, Alhoqail WA, Senousy HH (2022). Cyanobacteria-mediated immune responses in pepper plants against *Fusarium* wilt. Plants.

[CR4] Abdelaziz AM, El-Wakil DA, Attia MS, Ali OM, AbdElgawad H, Hashem AH (2022). Inhibition of *Aspergillus flavus* growth and aflatoxin production in *Zea mays* L. using endophytic *Aspergillus fumigatus*. J Fungi.

[CR5] Abdelaziz AM, Salem SS, Khalil A, El-Wakil DA, Fouda HM, Hashem AH (2022). Potential of biosynthesized zinc oxide nanoparticles to control *Fusarium *wilt disease in eggplant (*Solanum melongena*) and promote plant growth. Biometals.

[CR6] Abdel-Motaal F, Kamel N, El-Zayat S, Abou-Ellail M (2020). Early blight suppression and plant growth promotion potential of the endophyte *Aspergillus flavus* in tomato plant. Ann Agric Sci.

[CR7] Adnan M, Islam W, Shabbir A, Khan KA, Ghramh HA, Huang Z, Chen HY, Lu G-D (2019). Plant defense against fungal pathogens by antagonistic fungi with *Trichoderma* in focus. Microb Pathog.

[CR8] Alabouvette C, Olivain C, Migheli Q, Steinberg C (2009). Microbiological control of soil-borne phytopathogenic fungi with special emphasis on wilt-inducing *Fusarium oxysporum*. New Phytol.

[CR9] Aldinary AM, Abdelaziz AM, Farrag AA, Attia MS (2021). Biocontrol of tomato Fusarium wilt disease by a new Moringa endophytic *Aspergillus isolates*. Mater Today Proc.

[CR10] Alnusairi GS, Mazrou YS, Qari SH, Elkelish AA, Soliman MH, Eweis M, ElNahhas N (2021). Exogenous nitric oxide reinforces photosynthetic efficiency, osmolyte, mineral uptake, antioxidant, expression of stress-responsive genes and ameliorates the effects of salinity stress in wheat. Plants.

[CR11] Attia MS, El-Naggar HA, Abdel-Daim MM, El-Sayyad GS (2021). The potential impact of *Octopus cyanea* extracts to improve eggplant resistance against *Fusarium*-wilt disease: in vivo and in vitro studies. Environ Sci Pollut Res.

[CR12] Attia MS, El-Sayyad GS, Abd Elkodous M, Khalil WF, Nofel MM, Abdelaziz AM, Farghali AA, El-Batal AI, El Rouby WM (2021). Chitosan and EDTA conjugated graphene oxide antinematodes in Eggplant: toward improving plant immune response. Int J Biol Macromol.

[CR13] Attia MS, Abdelaziz AM, Al-Askar AA, Arishi AA, Abdelhakim AM, Hashem AH (2022). Plant growth-promoting fungi as biocontrol tool against *Fusarium* wilt disease of tomato plant. J Fungi.

[CR14] Attia MS, El-Wakil DA, Hashem AH, Abdelaziz AM (2022). Antagonistic effect of plant growth-promoting fungi against *Fusarium* wilt disease in tomato: in vitro and in vivo study. Appl Biochem Biotechnol.

[CR15] Attia MS, Hashem AH, Badawy AA, Abdelaziz AM (2022). Biocontrol of early blight disease of eggplant using endophytic *Aspergillus terreus*: improving plant immunological, physiological and antifungal activities. Bot Stud.

[CR16] Baaziz M (2011). Arbuscular mycorrhizal fungi limit incidence of *Fusarium oxysporum* f. sp. *albedinis* on date palm seedlings by increasing nutrient contents, total phenols and peroxidase activities. Open Hortic J.

[CR17] Badawy AA, Alotaibi MO, Abdelaziz AM, Osman MS, Khalil AM, Saleh AM, Mohammed AE, Hashem AH (2021). Enhancement of seawater stress tolerance in barley by the endophytic fungus *Aspergillus ochraceus*. Metabolites.

[CR18] Barceló AR, Muñoz R, Sabater F (1987). Lupin peroxidases. I. Isolation and characterization of cell wall-bound isoperoxidase activity. Physiol Plant.

[CR19] Bashan Y, De-Bashan LE (2010). How the plant growth-promoting bacterium *Azospirillum* promotes plant growth—a critical assessment. Adv Agron.

[CR20] Bates LS, Waldren RP, Teare I (1973). Rapid determination of free proline for water-stress studies. Plant Soil.

[CR21] Bergmeyer H (1974). Determination with glucose oxidase and peroxidase. Methods Enzym Anal.

[CR22] Bradford MM (1976). A rapid and sensitive method for the quantitation of microgram quantities of protein utilizing the principle of protein-dye binding. Anal Biochem.

[CR23] Büttner G, Pfähler B, Märländer B (2004). Greenhouse and field techniques for testing sugar beet for resistance to Rhizoctonia root and crown rot. Plant Breed.

[CR24] Chakraborty U, Chakraborty B, Basnet M (2006). Plant growth promotion and induction of resistance in *Camellia sinensis* by *Bacillus megaterium*. J Basic Microbiol.

[CR25] Chen X (2020). Pathogens which threaten food security: *Puccinia striiformis*, the wheat stripe rust pathogen. Food Security.

[CR26] Christ B, Hörtensteiner S (2014). Mechanism and significance of chlorophyll breakdown. J Plant Growth Regul.

[CR27] Ćilerdžić J, Vukojević J, Stajić M, Stanojković T, Glamočlija J (2014). Biological activity of *Ganoderma lucidum* basidiocarps cultivated on alternative and commercial substrate. J Ethnopharmacol.

[CR28] Cohen-Bazire G, Sistrom W, Vernon L, Seeley G (1966). The Chlorophylls.

[CR29] Daayf F, El Hadrami A, El-Bebany AF, Henriquez MA, Yao Z, Derksen H, El Hadrami I, Adam LR (2012). Phenolic compounds in plant defense and pathogen counter-defense mechanisms. Recent Adv Polyphen Res.

[CR30] Dai, G. H., Andary, C., Cosson-Mondolot, L., & Boubals, D. (1993, September). Polyphenols and resistance of grapevines to downy mildew. In International Symposium on Natural Phenols in Plant Resistance 381 (pp. 763-766). 10.17660/ActaHortic.1994.381.110

[CR31] Das K, Roychoudhury A (2014). Reactive oxygen species (ROS) and response of antioxidants as ROS-scavengers during environmental stress in plants. Front Environ Sci.

[CR32] De Rocchis V, Jammer A, Camehl I, Franken P, Roitsch T (2022). Tomato growth promotion by the fungal endophytes *Serendipita indica* and *Serendipita herbamans* is associated with sucrose de-novo synthesis in roots and differential local and systemic effects on carbohydrate metabolisms and gene expression. J Plant Physiol.

[CR33] Dhouib H, Zouari I, Abdallah DB, Belbahri L, Taktak W, Triki MA, Tounsi S (2019). Potential of a novel endophytic Bacillus velezensis in tomato growth promotion and protection against *Verticillium* wilt disease. Biol Control.

[CR34] Doohan Fiona, Zhou Binbin, Kevin Kavanagh (2017). Fungal pathogens of plants. Fungi: biology and applications.

[CR35] Elbasuney S, El-Sayyad GS, Attia MS, Abdelaziz AM (2022). Ferric oxide colloid: towards green nano-fertilizer for tomato plant with enhanced vegetative growth and immune response against *Fusarium* wilt disease. J Inorg Organomet Polym Mater.

[CR36] El-Beltagi HS, Mohamed AA, Rashed MM (2010). Response of antioxidative enzymes to cadmium stress in leaves and roots of radish (*Raphanus sativus* L/). Not Sci Biol.

[CR37] Elghaffar RYA, Amin BH, Hashem AH, Sehim AE (2022). Promising endophytic *Alternaria alternata* from leaves of *Ziziphus spina-christi*: phytochemical analyses, antimicrobial and antioxidant activities. Appl Biochem Biotechnol.

[CR38] Elmholt S (1996). Microbial activity, fungal abundance, and distribution of *Penicillium* and *Fusarium* as bioindicators of a temporal development of organically cultivated soils. Biol Agric Hortic.

[CR39] El-Tayeb MA (2005). Response of barley grains to the interactive effect of salinity and salicylic acid. Plant Growth Regul.

[CR40] Ferus P, Barta M, Konôpková J (2019). Endophytic fungus *Beauveria bassiana* can enhance drought tolerance in red oak seedlings. Trees.

[CR41] Ghaffari MR, Mirzaei M, Ghabooli M, Khatabi B, Wu Y, Zabet-Moghaddam M, Mohammadi-Nejad G, Haynes PA, Hajirezaei MR, Sepehri M (2019). Root endophytic fungus *Piriformospora indica* improves drought stress adaptation in barley by metabolic and proteomic reprogramming. Environ Exp Bot.

[CR42] Harb A, Krishnan A, Ambavaram MM, Pereira A (2010). Molecular and physiological analysis of drought stress in Arabidopsis reveals early responses leading to acclimation in plant growth. Plant Physiol.

[CR43] Hashem AH, Abdelaziz AM, Askar AA, Fouda HM, Khalil AM, Abd-Elsalam KA, Khaleil MM (2021). Bacillus megaterium-mediated synthesis of selenium nanoparticles and their antifungal activity against *Rhizoctonia solani* in Faba Bean Plants. Journal of Fungi.

[CR44] Hashem AH, Shehabeldine AM, Abdelaziz AM, Amin BH, Sharaf MH (2022). Antifungal activity of endophytic *Aspergillus terreus* extract against some fungi causing mucormycosis: ultrastructural study. Appl Biochem Biotechnol.

[CR45] Hibar K, Edel-Herman V, Steinberg C, Gautheron N, Daami-Remadi M, Alabouvette C, El Mahjoub M (2007). Genetic diversity of *Fusarium oxysporum* populations isolated from tomato plants in Tunisia. J Phytopathol.

[CR46] Hörtensteiner S, Kräutler B (2011). Chlorophyll breakdown in higher plants. Biochim et Biophys Acta BBA Bioenerg.

[CR47] Hu Z, Richter H, Sparovek G, Schnug E (2004). Physiological and biochemical effects of rare earth elements on plants and their agricultural significance: a review. J Plant Nutr.

[CR48] Hyakumachi M (2013). Research on biological control of plant diseases: present state and perspectives. J Gen Plant Pathol.

[CR49] Irigoyen J, Einerich D, Sánchez-Díaz M (1992). Water stress induced changes in concentrations of proline and total soluble sugars in nodulated alfalfa (*Medicago sativd*) plants. Physiol Plant.

[CR50] Jackson AO, Taylor CB (1996). Plant-microbe interactions: life and death at the interface. Plant Cell.

[CR51] Jagota S, Dani H (1982). A new colorimetric technique for the estimation of vitamin C using Folin phenol reagent. Anal Biochem.

[CR52] Kang SG, Lee KE, Singh M, Kumar P, Matin MN (2021). Rice lesion mimic mutants (LMM): the current understanding of genetic mutations in the failure of ROS scavenging during lesion formation. Plants.

[CR53] Khalil AMA, Hashem AH, Abdelaziz AM (2019). Occurrence of toxigenic *Penicillium polonicum* in retail green table olives from the Saudi Arabia market. Biocatal Agric Biotechnol.

[CR54] Khalil A, Abdelaziz A, Khaleil M, Hashem A (2021). Fungal endophytes from leaves of *Avicennia marina* growing in semi-arid environment as a promising source for bioactive compounds. Lett Appl Microbiol.

[CR55] Khan AL, Hamayun M, Kang SM, Kim YH, Jung HY, Lee JH, Lee IJ (2012). Endophytic fungal association via gibberellins and indole acetic acid can improve plant growth under abiotic stress: an example of *Paecilomyces formosus* LHL10. BMC Microbiol.

[CR56] Khattab AM, Abo-Taleb HA, Abdelaziz AM, El-Tabakh MA, El-Feky MM, Abu-Elghait M (2022). Daphnia magna and *Gammarus pulex*, novel promising agents for biomedical and agricultural applications. Sci Rep.

[CR57] Kim J, Campbell B, Mahoney N, Chan K, Molyneux R, May G (2007). Enhanced activity of strobilurin and fludioxonil by using berberine and phenolic compounds to target fungal antioxidative stress response. Lett Appl Microbiol.

[CR58] Maghsoudi K, Emam Y, Pessarakli M (2016). Effect of silicon on photosynthetic gas exchange, photosynthetic pigments, cell membrane stability and relative water content of different wheat cultivars under drought stress conditions. J Plant Nutr.

[CR59] Matta A, Dimond AE (1963). Symptoms of *Fusarium* wilt in relation to quantity of fungus and enzyme activity in tomato stems. Phytopathology.

[CR60] Mikulic-Petkovsek M, Schmitzer V, Jakopic J, Cunja V, Veberic R, Munda A, Stampar F (2013). Phenolic compounds as defence response of pepper fruits to *Colletotrichum coccodes*. Physiol Mol Plant Pathol.

[CR61] Morsy M, Cleckler B, Armuelles-Millican H (2020). Fungal endophytes promote tomato growth and enhance drought and salt tolerance. Plants.

[CR62] Mukherjee S, Choudhuri M (1983). Implications of water stress-induced changes in the levels of endogenous ascorbic acid and hydrogen peroxide in Vigna seedlings. Physiol Plant.

[CR63] Murray A, Kisin E, Castranova V, Kommineni C, Gunther M, Shvedova A (2007). Phenol-induced in vivo oxidative stress in skin: evidence for enhanced free radical generation, thiol oxidation, and antioxidant depletion. Chem Res Toxicol.

[CR64] Nefzi A, Abdallah RAB, Jabnoun-Khiareddine H, Ammar N, Daami-Remadi M (2019). Ability of endophytic fungi associated with *Withania somnifera* L. to control *Fusarium* Crown and Root Rot and to promote growth in tomato. Braz J Microbiol.

[CR65] Nisa H, Kamili AN, Nawchoo IA, Shafi S, Shameem N, Bandh SA (2015). Fungal endophytes as prolific source of phytochemicals and other bioactive natural products: a review. Microb Pathog.

[CR66] Olatunji TL, Afolayan AJ (2018). The suitability of chili pepper (*Capsicum annuum* L.) for alleviating human micronutrient dietary deficiencies: a review. Food Sci Nutr.

[CR67] Ownley BH, Gwinn KD, Vega FE (2010). Endophytic fungal entomopathogens with activity against plant pathogens: ecology and evolution. Biocontrol.

[CR68] Poveda J, Abril-Urias P, Escobar C (2020). "Biological control of plant-parasitic nematodes by filamentous fungi inducers of resistance: trichoderma, mycorrhizal and endophytic fungi. Front Microbiol.

[CR69] Rahman MA (2019). Rice (*Oryza sativa*) receptor for activated C kinase1b (OsRACK1B) regulates chlorophyll catabolism oxidative stress signaling and pollen development pathways. Biochemistry and Molecular Biology.

[CR70] Righini H, Roberti R, Varma A, Tripathi S, Prasad R (2019). Algae and cyanobacteria as biocontrol agents of fungal plant pathogens. Plant microbe interface.

[CR71] Rios-Gonzalez K, Erdei L, Lips SH (2002). The activity of antioxidant enzymes in maize and sunflower seedlings as affected by salinity and different nitrogen sources. Plant Sci.

[CR72] Rongai D, Milano F, Sciò E (2012). Inhibitory effect of plant extracts on conidial germination of the phytopathogenic fungus *Fusarium oxysporum*. American Journal of Plant Sciences.

[CR73] Sagi M, Davydov O, Orazova S, Yesbergenova Z, Ophir R, Stratmann JW, Fluhr R (2004). Plant respiratory burst oxidase homologs impinge on wound responsiveness and development in *Lycopersicon esculentum*. Plant Cell.

[CR74] Saia S, Corrado G, Vitaglione P, Colla G, Bonini P, Giordano M, Stasio ED, Raimondi G, Sacchi R, Rouphael Y (2021). An endophytic fungi-based biostimulant modulates volatile and non-volatile secondary metabolites and yield of greenhouse basil (*Ocimum basilicum* L.) through variable mechanisms dependent on salinity stress level. Pathogens.

[CR75] Sathiyabama M, Charles RE (2015). Fungal cell wall polymer based nanoparticles in protection of tomato plants from wilt disease caused by *Fusarium oxysporum* f. sp. *lycopersici*. Carbohyd Polym.

[CR76] Sharaf MH, Abdelaziz AM, Kalaba MH, Radwan AA, Hashem AH (2022). Antimicrobial, antioxidant, cytotoxic activities and phytochemical analysis of fungal endophytes isolated from *Ocimum basilicum*. Appl Biochem Biotechnol.

[CR77] Shigeoka S, Ishikawa T, Tamoi M, Miyagawa Y, Takeda T, Yabuta Y, Yoshimura K (2002). Regulation and function of ascorbate peroxidase isoenzymes. J Exp Bot.

[CR78] Snedecor G, Cochran WG (1980). Statistical methods.

[CR79] Sonawane H, Shelke D, Chambhare M, Dixit N, Math S, Sen S, Borah SN, Islam NF, Joshi SJ, Yousaf B (2022). Fungi-derived agriculturally important nanoparticles and their application in crop stress management–Prospects and environmental risks. Environ Res.

[CR80] Srinivas C, Devi DN, Murthy KN, Mohan CD, Lakshmeesha T, Singh B, Kalagatur NK, Niranjana S, Hashem A, Alqarawi AA (2019). *Fusarium oxysporum* f. sp. *lycopersici* causal agent of vascular wilt disease of tomato: Biology to diversity–A review. Saudi J Biol Sci.

[CR81] Strobel G (2018). The emergence of endophytic microbes and their biological promise. J Fungi.

[CR82] Supaphon P, Phongpaichit S, Rukachaisirikul V, Sakayaroj J (2013). Antimicrobial potential of endophytic fungi derived from three seagrass species: *Cymodocea serrulata*, *Halophila ovalis* and *Thalassia hemprichii*. PLoS ONE.

[CR83] Szabados L, Savouré A (2010). Proline: a multifunctional amino acid. Trends Plant Sci.

[CR84] Thipyapong P, Hunt MD, Steffens JC (1995). Systemic wound induction of potato (*Solanum tuberosum*) polyphenol oxidase. Phytochemistry.

[CR85] Van Loon L, Bakker P, Pieterse C (1998). Systemic resistance induced by rhizosphere bacteria. Annu Rev Phytopathol.

[CR86] Wani ZA, Ashraf N, Mohiuddin T, Riyaz-Ul-Hassan S (2015). Plant-endophyte symbiosis, an ecological perspective. Appl Microbiol Biotechnol.

[CR87] Yan L, Zhu J, Zhao X, Shi J, Jiang C, Shao D (2019). Beneficial effects of endophytic fungi colonization on plants. Appl Microbiol Biotechnol.

[CR88] Zou YN, Wu QS, Kuča K (2021). Unravelling the role of arbuscular mycorrhizal fungi in mitigating the oxidative burst of plants under drought stress. Plant Biol.

